# Patient Participation in Communication about Treatment Decision-Making for Localized Prostate Cancer during Consultation Visits

**DOI:** 10.4236/health.2015.711156

**Published:** 2015-11-11

**Authors:** Lixin Song, Mark P. Toles, Jinbing Bai, Matthew E. Nielsen, Donald E. Bailey, Betsy Sleath, Barbara Mark

**Affiliations:** 1 School of Nursing, University of North Carolina (UNC), Chapel Hill, USA; 2 Lineberger Comprehensive Cancer Center, University of North Carolina (UNC), Chapel Hill, USA; 3 Department of Urology, School of Medicine, University of North Carolina (UNC), Chapel Hill, USA; 4 School of Nursing, Duke University, Durham, USA; 5 Eshelman School of Pharmacy, University of North Carolina, Chapel Hill, USA

**Keywords:** Localized Prostate Cancer (LPCa), Decision-Making, Patient-Provider Communication, Patient Participation, Audio-Recording, Consultation

## Abstract

**Objectives:**

To describe the communication behaviors of patients and physicians and patient participation in communication about treatment decision-making during consultation visits for localized prostate cancer (LPCa).

**Methods:**

This is a secondary analysis of data from 52 men enrolled in the usual care control group of a randomized trial that focused on decision-making for newly diagnosed men with LPCa. We analyzed the patient-physician communication using the transcribed audio-recordings of real-time treatment consultations and a researcher-developed coding tool, including codes for communication behaviors (information giving, seeking, and clarifying/ verifying) and contents of clinical consultations (health histories, survival/mortality, treatment options, treatment impact, and treatment preferences). After qualitative content analysis, we categorized patient participation in communication about treatment-related clinical content, including “none” (content not discussed); “low” (patient listening only); “moderate” (patient providing information or asking questions); and “high” (patient providing information and asking questions).

**Results:**

Physicians mainly provided information during treatment decision consultations and patients frequently were not active participants in communication. The participation of patients with low and moderate cancer risk typically was: 1) “moderate and high” in discussing health histories; 2) “low” in discussing survival/mortality; 3) “low and moderate” in discussing treatment options; 4) “none and low” in discussing treatment impacts; and 5) “low” in discussing treatment preferences.

**Conclusions:**

Findings suggest opportunities for increasing patient participation in communication about treatment decision-making for LPCa during clinical consultations.

## 1. Introduction

Communication between the healthcare provider (HCP) and patient is essential in helping HCPs to understand and address patient concerns and information needs, and ultimately, promoting shared decision-making and patient quality of life (QOL) [1]. During patient-centered clinical encounters, patients share the values and concerns about the potential benefits and harms of different treatment options; during the consultations, HCPs explain the medical condition and treatments [1]–[3] and help patients make informed treatment decisions by comparing treatment options with similar outcomes but different side-effects [4]. Patient-HCP communication varies from one-way communication *(i.e.*, information flow from HCP to patient with limited patient involvement) to two-way communication *(i. e*., a patient-provider partnership in which patients share the power, responsibility, and their preferences and values) [5]. During patient-HCP interactions in clinical consultations, information giving, seeking, clarifying and verifying are important communication behaviors that help patients and HCPs promote information exchange (i.e., patient education and patient participation) between HCPs and patients [6]. Studies indicate that patient-HCP communication is associated with treatment decision-making among cancer patients [7] [8], and ultimately, patient satisfaction with healthcare services [9] and health outcomes [1] [7] [8].

Because no one treatment strategy is clearly superior in terms of mortality for treating localized prostate cancer (LPCa), decision-making about treatments for LPCa should be based on patient values and preferences, while accounting for tradeoffs between the harms and benefits of treatment options (e.g., surgery, radiation, hormonal therapy, active surveillance/watchful waiting) [10]–[12]. The goal of treatment for LPCa is not always curative; it is also to ensure that patients experience the best QOL after treatment. Thus, patient-HCP communication is critical in helping patients understand potential treatment options and their impacts, which facilitates appropriately informed decision-making for patients [8]. Research has shown that patients often choose a treatment due to their lack of awareness of alternatives and have unrealistic expectations about possible treatment outcomes [13] [14]. For example, up to a third of patients with LPCa report decisional regrets when their QOL deteriorates and/or treatment side-effects have negative impacts on their lives after treatment [15] [16]. These unwanted outcomes may be due to patients’ less active participation in patient-HCP communication during treatment consultations.

However, studies of patient participation in communication about treatment decision-making for LPCa during consultation visits are limited. Most studies used data collected from patients after treatment decisions were made [9] [17], which increased the risk for recall [18] and social desirability bias [19]. In contrast, analysis of real-time consultation interviews captures discussions of treatment options and/or when treatment decisions are reached. For example, using audio-recorded transcripts, Henry and colleagues analyzed the overall structure of clinical visits *(i.e.*, the sequence of and transitions between patient-physician communication activities) when physicians discussed diagnosis and treatment with patients with newly diagnosed LPCa [20]. They found physicians focused on discussing treatment options and devoted little time and attention to discussing the new cancer diagnosis. However, this study only analyzed patient-physician communication activities (e.g., diagnosis delivery, risk classification, options talk, decision talk and next steps) based on the approximate time spent on each activity and the linguistic features such as topic shifts and discourse markers (e.g., use of “well”, “oh”), rather than on who was speaking. Moreover, the study did not include either an analysis of the specific communication behaviors of patients and HCPs related to the detailed content of patient-physician communication or patient participation in communication about treatment decisions. Thus, whether and how patients and providers used the communication behaviors (e.g., information providing, seeking, and clarifying) in exchanging information about specific topics about treatment decision-making during consultation visits have not been richly elaborated and detailed.

The purpose of this study was to analyze specific patient and physician communication behaviors (information giving, seeking, clarifying and verifying) and describe patient participation in patient-provider communication about the contents of LPCa treatment decision-making during consultation interviews. We defined patient-provider communication as the dynamic, interpersonal process of mutual influence that occurs during the verbal exchange of information between physician and patients [8] [21]. The expected outcome of the study was data about the context of LPCa treatment decision-making that can be used to help patients understand their diagnosis, offer them different treatment options, answer questions about the potential side effects of treatment options, and explore patients’ values and preferences as they make decisions about next steps in their cancer treatment [21].

## 2. Methods

### 2.1 Subjects

This is a secondary analysis of the transcribed audio-recorded real-time consultation interviews with control subjects in the usual care group of a randomized clinical trial that tested the effects of an intervention designed to improve informed treatment decision-making for patients with LPCa [22]. In the trial, conducted between 2004 and 2008, patients were eligible if they 1) were newly diagnosed with LPCa (stages T1a, b, c or T2a or T2b); 2) were at least 10 days before the treatment consultation appointment; 3) had no major cognitive impairment; 4) had no prior cancer history; and 5) could read and speak English. Among 410 men contacted, 343 were eligible, and 256 agreed to participate in the study [22]. Patients were randomized into 3 groups: a control group with usual care (control), intervention directed to the patient (TD), and intervention directed to the patient and family support person (TS). The intervention presented communication strategies through a DVD, a booklet that provided a patient-focused, evidence-based guide to treatment issues for early stage PCa, and 4 telephone calls to the subject by a trained nurse interventionist. The patients and family members in the TS group received the 4 telephone calls separately from the same nurse. To reduce impacts of the intervention on patient-physician communication behaviors, only patients from the control group were included in this study.

The purpose of the consultations was for patients to discuss treatment options and seek support for treatment decision-making after receiving their biopsy results and diagnosis. Consultation visits were audio-recorded and transcribed verbatim. Physicians in the original study were blinded to the participants by the taping of an equal number of non-study patients. Physicians also agreed to place a sign in their clinics announcing that patient-physician communication would be randomly taped in order to inform their patients the purpose of the recording *(i.e.*, to verify the length of their interviews). Non-study patients were also newly diagnosed but had refused to be in the randomized trial or did not meet study criteria; they were approached and consented for the recoding of random interviews; and their interview tapes were erased after their sessions. Details about the study sample and procedures were reported previously [22]. Approval was obtained from the Institutional Review Boards at all study sites.

### 2.2 Measurement

1. Patient characteristics measures.
  Patient age and number of years of education were measured as continuous variables. Self-reported categorical variables included race, marital status, monthly family income, full time working status, and health insurance. Cancer information *(i.e.*, Gleason score and prostate-specific antigen, PSA) was reported by patients and verified by physicians during the clinical encounter.
2. Communication measures.
  The communication measures were developed by the research team (LS, BB & BS) based on the National Comprehensive Cancer Network (NCCN) treatment guideline for LPCa [23] and medical consultation [21], theories on shared decision-making [24] and previous research on patient-HCP communication [25] [26]. As displayed in Table 1, the measures included codes for communication behaviors of patients and their physicians and content of the consultation visits he communication behaviors included information giving, clarifying/ verifying, and seeking. Consultation content included 5 domains a) patient health histories *(i.e.*, cancer diagnosis, current LPCa-related symptoms, and comorbid condition); b) treatment options *(i.e.*, surgery, radiotherapy, watchful waiting/active surveillance, and hormonal therapy); c) potential treatment impact *(i.e.*, complications; impacts on QOL; urinary, sexual, bowel and hormonal side-effects; and management of side-effects); d) treatment-related survival/mortality and e) treatment preferences.

Using the codes for communication behaviors and contents of consultation visits, we conducted content analysis of transcripts from the consultation visits. Two coders (LS and JB) separately coded whether the communication behaviors related to each content category were or were not observed *(i.e.*, coded as “Yes” and “No”, respectively). The reliability of the coding was evaluated in a random sample of 25% of the consultation transcripts; the agreement between the two coders was 0.87 and 0.90 for patient and physician communication behaviors across consultation content domains, respectively, supporting high inter-rater reliability [27].

Although more than one physician was involved during some consultation visits, only one physician was in the room with the patient at a time. In order to capture all the communication behaviors used by physicians, we coded the behaviors of all physicians as if they were one single interview with one physician. The decision to combine across interviews was based on the consideration that physicians might discuss the same communication content (as listed above) using different communication behaviors to help patients understand their treatment options and make informed decisions during a consultation.

To examine patient participation in communication during consultations, we conducted a secondary thematic analysis using cross tabulation to link a patient’s communication behaviors within each content category with those of his physician(s) (Note: information seeking and information clarifying/verifying were collapsed due to their infrequent usage). As displayed in Table 2, we identified 4 categories of patient participation that represented instances in which the patient passively received information from physicians to instances in which the patient actively provided information and asked questions. Patient participation in communication about each content category were classified as none (specific content domain not discussed); low (patient listened/passively received information as demonstrated by physician information giving only and the patient exhibited **no** verbal communication behaviors directed toward the physician); moderate (patient-physician interaction as demonstrated by patient information giving **or** information clarifying/seeking); and high (patient actively interacting with physician as demonstrated by patient information giving **and** clarifying/seeking).

### 2.3. Data Analysis

Univariate analysis was conducted to describe the counts of communication behaviors within content categories separately for patients and physicians. Descriptive analysis was used to examine patient participation in communication for each content category according to patients’ prostate cancer risk level because treatment options differ by risk levels [23]: low (Gleason ≤ 6 or PSA < 10.0 ng/ml), intermediate (Gleason = 7 or PSA ≥ 10 but < 20 ng/ml), and high risk (Gleason ≥ 8 or PSA ≥ 20 ng/ml) [23].

## 3. Results

### 3.1. Participants

The audio-recorded treatment consultation interviews were from 52 patients. Table 3 presents patient demographics. Participants were African-Americans (N = 16) and Caucasians (N = 36). On average, patients were 60.2 years of age and had 16 years of education. Forty-two patients were married or partnered; twenty-seven reported a monthly family income greater than $4000; and 64% had low risk prostate cancer. Forty-two patients had consultations provided by one physician; 11 patients had a consultation provided by 2 to 3 physicians (and/or residents and fellows). Fifty-nine physicians, 3 residents and 1 fellow were involved in these consultations. Among these physicians, 50 were urologic surgeons, 7 radiation oncologists, and 2 medical oncologists.

Regarding treatment decisions, 32% of the patients had made a tentative decision before attending their consultations; 79% received treatment recommendations from the physicians during the consultation. About 65% were given additional time for decision-making *(i.e.*, allowed to leave the consultation without a treatment plan).

### 3.2. Patient and Physician Communication Behaviors

**Figure 1.**
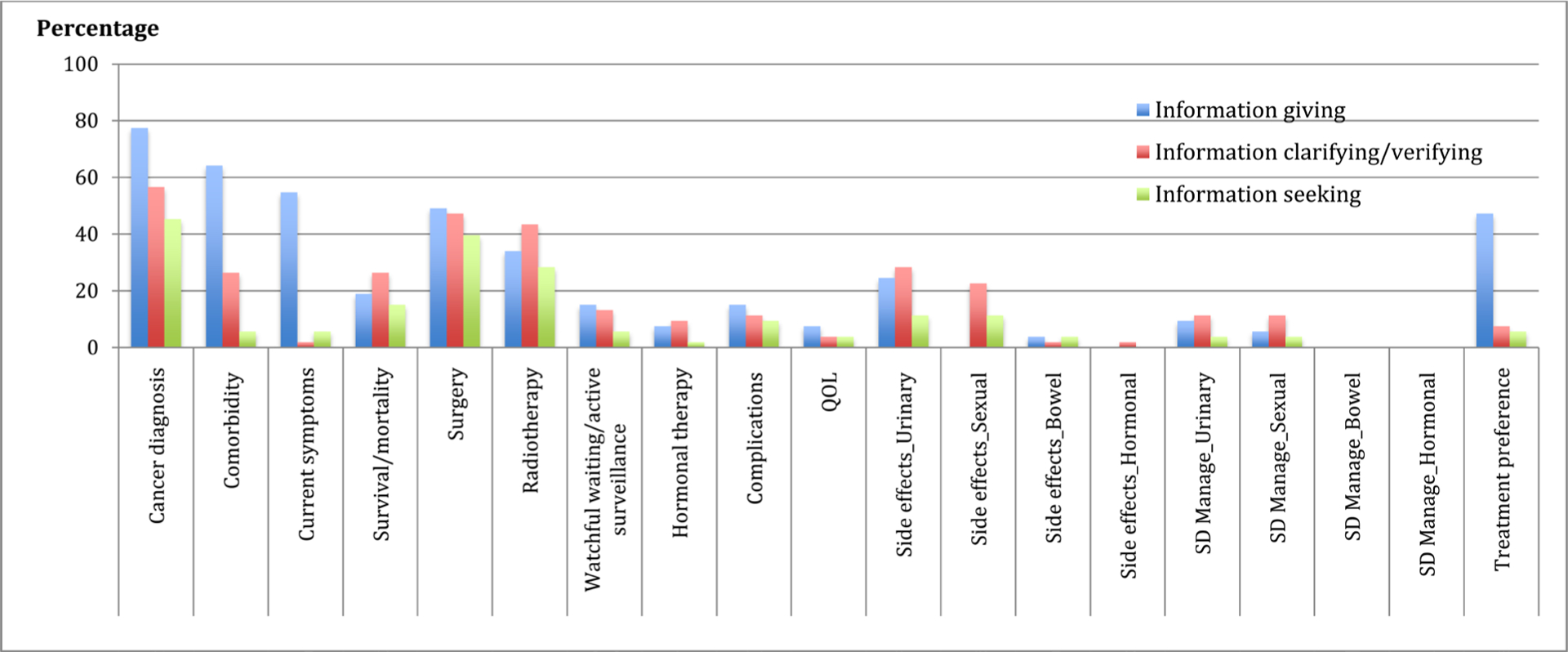
Patient communication behaviors. **Note:** D1 = patient health history (i.e., cancer diagnosis, current LPCa-related symptoms, and comorbid condition); D2 = treatment options (i.e., surgery, radiotherapy, watchful waiting/active surveillance, and hormonal therapy); D3 = potential treatment impact (i.e., complications; impacts on QOL; urinary, sexual, bowel and hormonal side-effects; and management of side-effects); D4 = treatment-related survival/mortality; and D5 = treatment preferences.

Patient communication behaviors are displayed in Figure 1 (N = 52 transcripts/patients). More than half of the patients engaged in information giving, clarifying/verifying, and seeking behaviors in discussing their health histories, survival/mortality, treatment options of surgery and radiotherapy, potential treatment impact such as urinary and sexual side-effects, and treatment preferences. In contrast, only 25% to 50% of the patients demonstrated these communication behaviors in discussing treatment options related to watchful waiting/active surveillance or hormonal therapy, treatment impact (complications, QOL, bowel and hormonal side-effects), and management of side-effects.

**Figure 2.**
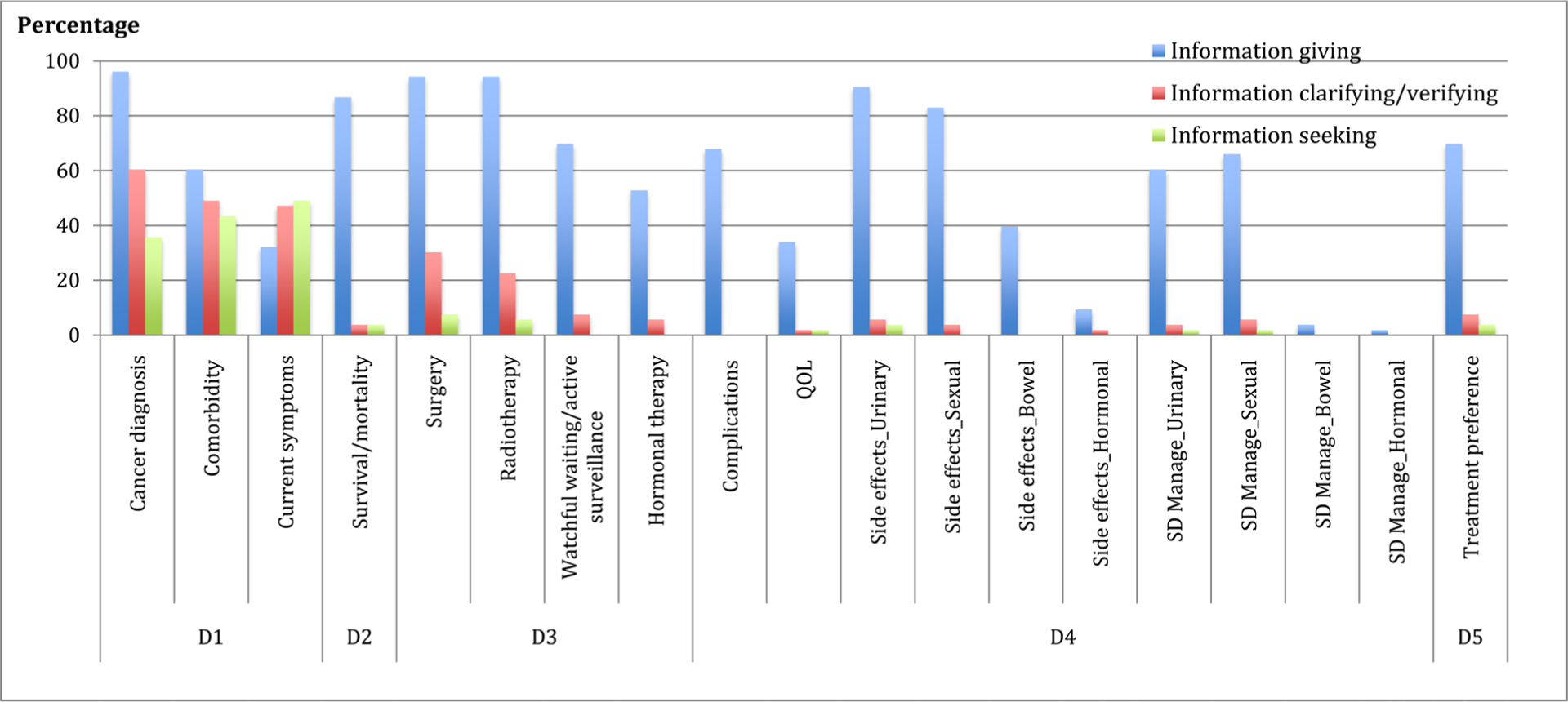
Physician communication behaviors. **Note:** D1 = patient health history (i.e., cancer diagnosis, current LPCa-related symptoms, and comorbid condition); D2 = treatment options (i.e., surgery, radiotherapy, watchful waiting/active surveillance, and hormonal therapy); D3 = potential treatment impact (i.e., complications; impacts on QOL; urinary, sexual, bowel and hormonal side-effects; and management of side-effects); D4 = treatment-related survival/mortality; and D5 = treatment preferences.

As displayed in Figure 2 (N = 52 transcripts/patients), physicians used more information giving than information clarifying and seeking across all consultation content domains. Information giving was used in more than 50% to 80% of the consultations when discussing all content domains except current symptoms, quality of life issues, bowel and hormonal side-effects, and the management of these side-effects. Physicians used information clarifying and seeking behaviors in over 90% of the consultations when discussing patient’s health histories; in more than 40% of the consultations when discussing treatment options related to surgery and radiotherapy; and in less than 10% consultations when discussing treatment options related to watchful waiting/active surveillance and hormonal therapy, treatment impact, and treatment preferences.

### 3.3. Patient Participation in Communication about Treatment Decision-Making

**Figure 3.**
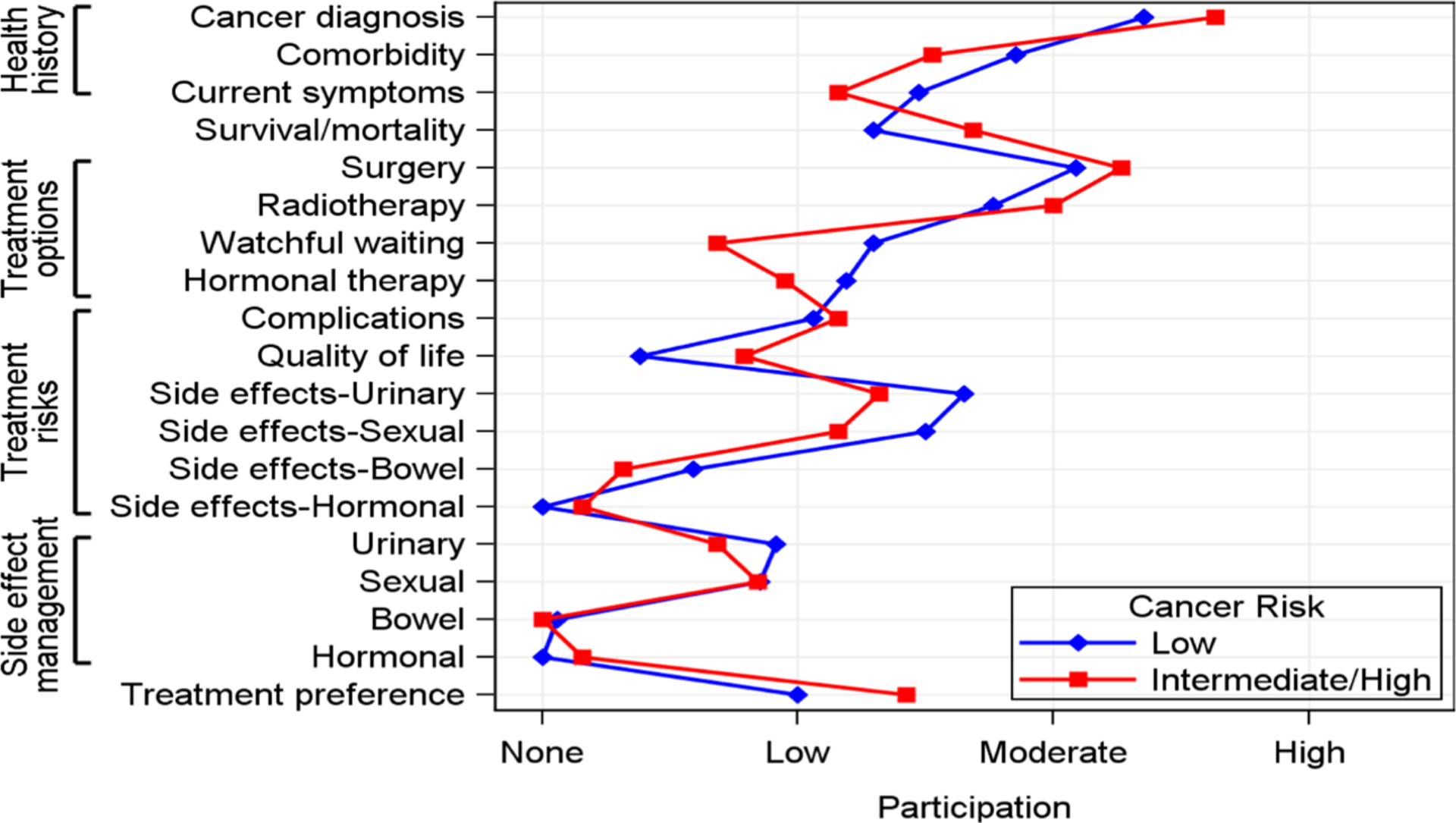
Patient participation in communication about treatment decision-making during consultation interviews.

Figure 3 displays patient participation in communication about treatment decision-making. Due to the small number in the high-risk group (N = 2), patients with high and intermediate risks were combined into one group. Patients with low and intermediate prostate cancer risk demonstrated similar participation patterns. First, patient participation ranged from low to high in discussing their health histories. Patient participation was moderate in discussing cancer diagnoses and comorbid conditions but low in discussing current prostate cancer-related symptoms. Second, patient articipation was low in discussing survival and mortality issues. Third, regarding treatment options, patient participation was moderate in discussing surgery and radiotherapy and low in discussing hormonal therapy and active surveillance/watchful waiting.

Patient participation was none or low when discussing potential treatment impacts. Patient participation was low when discussing complications, QOL issues, urinary and sexual side-effects, and management of urinary and sexual side-effects, whereas hormonal and bowel side-effects and management of these side-effects were not discussed. Finally, patient participation was low in discussing their treatment preferences.

## 4. Discussion

Patient-physician communication is integral to information exchange and treatment decision-making [4] [13]. Understanding patient-physician communication and patient participation in communication during consultations about treatment decision-making for LPCa is thus important to clinical practice. This study used audio-recordings of real-time consultation interviews to analyze patient participation in patient-provider communication about treatment decision-making for LPCa. Our findings show that, in the majority of consultation visits, communication consisted of physicians providing information, whereas patients’ communication behaviors varied across different consultation content categories. We also found patients with low and intermediate LPCa risks had similar patterns of participation in communication; they minimally identified or conveyed their treatment goals and concerns during the consultations.

We found that patients had none or low participation in discussing survival/mortality, treatment-related complications, QOL issues, side-effects, management of side-effects, and treatment preferences. Patients had moderate or high participation in discussing content related to their health histories. This is consistent with findings from previous research in which physicians engaged in significantly more exploration of patients’ disease and illness, but did not consistently engage in understanding the whole person [28]. A possible explanation for these findings might be that personal health histories were more familiar to the patients but new to physicians that patients met for the first time after their LPCa diagnosis. Alternatively, some patients may have been less familiar with topics such as treatment options, treatment impact on QOL, side-effect, and management of side-effects. In these consultations, when patients were less able or comfortable asking questions, their physicians may have engaged in more information giving. In this context, when physicians did not bring up certain topics (e.g., bowel and hormonal related side-effects, active surveillance/watchful waiting), patients received no or limited related information. Our findings suggest that patient participation in some content categories could be improved to facilitate the complex, preference-sensitive treatment decision-making for LPCa during consultation interviews.

In addition, it is unclear whether physicians incorporated patients’ preferences in treatment decision-making consultations. Despite limited patient participation in communication, 79% of the patients received treatment recommendations and about half made a treatment decision before leaving the consultation. It is possible that patients relied on the expertise of their physicians and believed that the best choice was their physicians’ opinions, rather than their own beliefs about treatment outcomes. Many patients asked limited or no questions using information seeking and clarifying during their consultation visits; thus, they may have missed opportunities for informed decision-making. Moreover, low patient participation in communication about some treatment decision-making content categories (e.g., watchful waiting/active surveillance and treatment impacts) suggests that patients might have received a treatment recommendation and/or made a treatment decision without fully understanding of all reasonable alternatives and/or the pros and cons of those options. Experimental research with informing patients facing surgical decisions found that more informed patients opted for more “conservative” treatment options [12]. Among patients with LPCa, lack of active participation in the decision-making process has been related with post-treatment decisional regret [29].

Finally, our findings suggest potential strategies to improve patient participation in communicating about treatment decision-making for LPCa. Healthcare providers have been identified as the major source of cancer information, especially among older adults [30]. Physicians usually set the agenda of clinical encounters, and thus, have the responsibility to encourage patients to participate by presenting treatment alternatives and their impacts as well as by asking questions to ensure patients fully understand the information they provide. On the other hand, patients may benefit from receiving treatment related information before consultations so that they can prepare questions in advance. Interventions can also enhance patient communication skills (e.g., teaching patients that it is ok to ask questions, how to ask questions, how to obtain additional information and how to clarify or verify understanding). These strategies will likely empower patients to clarify misunderstandings and to vocalize their values and preferences, and ultimately, to prepare patients to choose treatments guided by facts and realistic expectations.

This study had limitations. First, some of our audio recordings are nearly 10 years old and there have been considerable changes since that time in treatment options and decisions support available to men with LPCa (e.g., the Internet and support systems other than HCPs opened new venues for obtaining information to make decisions [31]). All of these factors, coupled with the movement toward patient activation and involvement [3], may have made cancer patients more active consumers of health care systems and shaped the communication of treatment decisions, options, alternatives, and the complications. Our research, however, is still relevant to today’s understanding of treatment decision-making related communication given the fact that older adults still rely heavily on medical professionals for information needed to make diagnostic or standard treatment decisions [32] [33]. More research is needed to examine whether patient participation in communication during consultation visits has changed as patient-HCP communication has become more complex given their access to information from more diverse sources and use of computers during consultation visits. Second, this was a secondary analysis of data from an earlier study, and thus, information such as the length of visits and physicians’ demographics were not available. Future research should include this information because it affects patient-physician communication (e.g., length and in-depth of discussion of contents). And also, due to the small sample size of non-urologists, the differences in behaviors among the specialists were not examined. Next, we focused on patient participation in patient-HCP communication; thus, family members’ involvement in communication was not included in this report. Finally, patient preference for and satisfaction with their participation were not assessed. Future research should identify patient communication style and preference so that communication can be more tailored to meet patient needs for participation in treatment decision-making related communication [13].

## 5. Implications for Practice

We analyzed patient participation in communication about treatment decision-making for LPCa using audio-recordings of real-time consultation visits. While physicians mainly used information providing, we found that patient participation was less than optimal in some decision-making related content categories. Our findings also suggest that, to improve patient participation, HCPs may integrate strategies such as physicians asking questions to ensure patient understanding of the information provided, providing patients with information about treatment prior to the consultation and/or communication skills *(i.e.*, decision aids [34]).

## Figures and Tables

**Table 1.** Coding tool for analyzing the consultation interview for treatment decision-making for LPCa.

CODING DIMENSIONS	DEFINITION
**Communicators/participants**	The patient and his physician(s)
**Communication Behaviors**	
Information giving	The unidirectional transmission of information from the patient to his physician(s) or vice versa, as demonstrated by the use of statements and presentation of facts.
Information seeking	The action of redirecting the communication content/topic by using a question or a statement
Information clarifying/verifying	The action of making information less confused and uncertain but more comprehensible by negotiating, confirming between multiple possibilities, and obtaining additional information.
**Communication Contents**	
Patient’s health history	Includes patient’s diagnosis, LPCa-related symptoms and comorbid conditions
Treatment options	Includes surgery (e.g., radical prostatectomy, robotic surgery), radiotherapy (e.g., internal versus external), watchful waiting or active surveillance, and hormonal therapy.
Treatment Impact	Includes treatment related complications (e.g., bleeding), impact on the patient’s QOL, treatment-related side-effects *(i.e.*, urinary, sexual, bowel, and hormonal dysfunction), and strategies for managing side-effects.
Survival/mortality	The 5- and/or 10-year survival statistics and/or the number of deaths related to LPCa and/or treatment for it.
Treatment preference	The value(s) that the patient attaches to any aspects of the treatment options

**Table 2.** Dyadic coding system for patient participation.

		Patient		Physician(s)	
Patient participation	Information flow between the patient and his physician(s)	Information giving	Information clarifying/seeking	Information giving	Information clarifying/seeking
None	Specific topic not discussed (No information flow between patient and physician)	No	No	No	No
Low	One-way flow from physician(s) to patient	No	No	Yes	No
Moderate [1]	Limited two way flow between patient and physician(s)	Variable (Yes or No)	Variable	Variable	Variable
High [2]	Active two way flow between patient and physician(s)	Yes	Yes	Variable	Variable

Note: the separate codes of information seeking and clarifying/verifying were collapsed into information clarifying/seeking in data analysis due to their very low frequency use during the consultations.

[1] for moderate participation, patients demonstrated **either** information giving **or** information clarifying/seeking behaviors, but not both at the same time.

[2] for high participation, patients had to demonstrate **both** information giving **and** information clarifying/seeking behaviors at the same time.

**Table 3.** Participant demographic characteristics.

Variable	Sample Size	Mean (SD), Range	N (%)
General Information			
Age	51	60.2 (7.1), 45 – 73	
Education	51	15.8 (3.4), 8 – 23	
Ethnicity	52		
Caucasian			36 (69.8)
African-American			16 (30.2)
Marital status	51		
Married/partnered			42 (82.4)
Single/widowed			9 (17.6)
Monthly family income	48		
$500 – 1000			5 (10.4)
$1001 – 2000			5 (10.4)
$2001 – 4000			11 (22.9)
> $4000			27 (56.3)
Full-time working status, Yes	48		38 (79.2)
Health insurance coverage, Yes	51		50 (94.3)
Decision-related Information*			
Decision-made before visit, Yes	50		16 (32.0)
Recommended treatment, Yes	48		38 (79.2)
Decision-made when left, Yes	49		24 (49.0)
Additional time for decision, Yes	48		31 (64.6)
**Disease-related Information**			
Gleason Score	46	6.37 (0.61), 5 – 8	
≤6			28 (60.9)
=7			17 (37.0)
≥8			1 (2.2)
Prostate Specific Antigen	48	8.00 (7.19), 3 – 40	
Cancer risk level	52		
Low			34 (64%)
Intermediate			17 (32%)
High			1 (4%)

* Items are not mutually exclusive, and thus, the total % is greater than 100.
